# Comprehensive management of chest wall recurrent HER2-positive breast cancer with Anlotinib and hypofractionated radiotherapy: a case report

**DOI:** 10.3389/fonc.2025.1708884

**Published:** 2026-01-05

**Authors:** Jing Xu, Qing Liu, Yue Yin, Guanglu Dong

**Affiliations:** 1 Department of Oncology and Radiotherapy, the Second Affiliated Hospital of Harbin Medical University, Harbin, Heilongjiang, China; 2 Harbin Medical University, Harbin, Heilongjiang, China

**Keywords:** Anlotinib, chest wall recurrent breast cancer, conventional fractionated radiotherapy, hypofractionated radiotherapy, trastuzumab

## Abstract

HER2-positive breast cancer (BC) patients who have developed resistance to various systemic therapies face limited treatment alternatives. Literature on Anlotinib combined with hypofractionated radiotherapy (HFRT) for recurrent/metastatic HER2-positive BC is scarce, particularly for Trastuzumab-resistant cases. This report describes an unresectable, bulky HER2-positive BC recurrence in a patient who had an inadequate response to multiple systemic treatments, including chemotherapy, Trastuzumab, immunotherapy, and conventional fractionated radiotherapy (CFRT). Upon observing further progression of the chest wall mass, we initiated a treatment regimen that combined Anlotinib with HFRT. Remarkably, after just one cycle of this combined therapy, the tumor exhibited considerable shrinkage, with the patient now remaining in remission. These findings indicate that Anlotinib, in conjunction with HFRT, may be a promising option for patients with unresectable chest wall recurrence(CWR), particularly for those who have previously undergone Trastuzumab therapy but subsequently developed resistance.

## Introduction

BC ranks as the most common malignant tumor affecting women worldwide (1–3). Fortunately, over recent decades, the outlook for BC patients has greatly improved, thanks to enhancements in screening techniques and a holistic approach to treatment. Various therapies, including surgery, chemotherapy, radiotherapy, endocrine therapy, targeted therapy, and immunotherapy, are vital at different stages of treating BC. In BC management, radiotherapy serves as a key treatment method, frequently utilized for patients who have had mastectomy, breast-conserving surgery, and those with inoperable cases. Employing adjuvant radiotherapy after breast-conserving surgery has been shown to significantly lower the rates of local recurrence and cancer-related deaths (4). Moreover, in patients receiving modified radical mastectomy, adjuvant radiotherapy can improve both local recurrence-free survival and overall survival rates (5, 6).As a result, various guidelines for BC advocate for postoperative radiotherapy as a crucial form of adjuvant treatment, especially for individuals with larger tumors and/or lymph node metastasis. Among BC patients who have undergone mastectomy, CWR is the most prevalent form of local recurrence (7). This typically presents as noticeable abnormal masses that consistently grow and feel firm, along with the presence of multiple lymph nodes in the skin and subcutaneous areas. Radiotherapy acts as a powerful local intervention for minimizing the risk of BC recurrence and reducing mortality rates (8). Standard adjuvant radiotherapy applied to the chest wall following mastectomy generally involves daily doses of 2 Gy, delivered over a period of 5–6 weeks (CFRT). Biological models of HFRT using doses greater than 2 Gy show comparable effectiveness to standard treatment regimens while decreasing the number of fractions and total treatment duration (9). Findings from extensive randomized studies involving women who received whole-breast radiotherapy post-breast-conserving surgery reveal that the effectiveness and long-term effects of HFRT are similar to those observed with CFRT (10–13). Additionally, HFRT has been implemented for patients needing adjuvant radiotherapy after mastectomy, particularly in regions where radiotherapy resources are scarce, aiming to shorten patient wait times and improve patient flow (14).

The HER2 is often overexpressed in BC, making anti-HER2 targeted therapy a fundamental aspect of treatment for HER2-positive cases. When Trastuzumab is combined with chemotherapy, it significantly enhances the pathological complete response (pCR) rate compared to chemotherapy alone, thereby positioning Trastuzumab as the standard care for HER2-positive BC. The U.S. Food and Drug Administration has authorized Trastuzumab for use in metastatic or recurrent disease and it is also utilized as postoperative or adjuvant therapy in early-stage BC (15). Anlotinib, an oral multi-target tyrosine kinase inhibitor, targets various signaling pathways such as the vascular endothelial growth factor receptor (VEGFR), platelet-derived growth factor receptor (PDGFR), and fibroblast growth factor receptor (FGFR), which can hinder tumor growth and angiogenesis (16). While its primary action is not specifically aimed at HER2, its influence on the tumor microenvironment and capacity to inhibit cancer cell proliferation suggest it may be a viable treatment alternative for HER2-positive BC.

To the best of our understanding, the effectiveness of Anlotinib in BC that is HER2-positive remains uncertain. Nonetheless, clinical studies have suggested that Anlotinib might show efficacy in specific patients with HER2-positive BC exhibiting well-characterized drug resistance. Additionally, there is a scarcity of information regarding the efficacy and safety of Anlotinib when used in conjunction with HFRT for recurrent or metastatic HER2-positive BC. From a biological standpoint, the theoretical basis for the synergistic use of Anlotinib and radiotherapy is well-established. Tumors often exhibit a disorganized vascular network that leads to inconsistent blood flow and creates a hypoxic microenvironment. This hypoxia can contribute to tumor cell resistance to radiation through various molecular routes, including the activation of the HIF-1α signaling pathway, and promotes the enhancement of DNA repair mechanisms (17, 18). Anlotinib, a multi-targeted tyrosine kinase inhibitor, markedly aids in normalizing tumor vasculature by selectively blocking receptors like VEGFR2/3, PDGFR-β, and FGFR1-4, which consequently boosts local blood flow and improves oxygen partial pressure (19). Research conducted prior to clinical trials has shown that antiangiogenic therapies can elevate the oxygen enhancement ratio (OER) in radiotherapy by decreasing tumor interstitial pressure, thus providing substantial theoretical backing for this combination treatment (18–20). Furthermore, agents like Anlotinib have been observed to ‘normalize’ the vasculature of tumors within a designated time frame, which leads to a temporary enhancement of blood flow and oxygen levels, therefore reducing hypoxia (21).HFRT destroys abnormal tumor vasculature, while Anlotinib normalizes functional vessels. Radiotherapy may trigger pro-angiogenic responses (e.g., VEGF upregulation), which Anlotinib can counteract (22). These mechanisms offer a strong theoretical rationale for combining HFRT with Anlotinib.

In this document, we present the case of a 59-year-old woman diagnosed with ER- and PR-, HER2-positive recurrent metastatic BC. She received neoadjuvant combination therapy consisting of carboplatin and albumin-bound paclitaxel alongside Toripalimab for four cycles before undergoing radical surgery, which was followed by a change to cyclophosphamide and doxorubicin for three cycles. After surgery, a recurrence on the chest wall was observed, prompting eight cycles of Trastuzumab treatment. Due to an inadequate response from the tumor, CFRT was later utilized for local management; however, the tumor continued to progress. Following five sessions of HFRT paired with Anlotinib treatment, the recurrent lesion on the chest wall showed a significant reduction and is presently in remission.

## Case report

**Figure 1:**
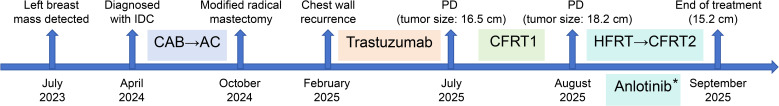
Timeline of treatment history and clinical course. The upper panel illustrates key clinical events and tumor measurements. A 3 cm left breast mass was diagnosed as invasive ductal carcinoma (IDC), grade III, by core needle biopsy in April 2024. Following a modified radicaly 2025 (HER2 IHC 3+, ~4 cm). The lower panel details the therapeutic regimens and corresponding responses: neoadjuvant chemotherapy (CAB/AC, Apr-Oct 2024) resulted in a partial response (PR); subsequent Trastuzumab therapy (Feb-Jul 2025) led to progressive disease (PD). Radiotherapy Phase 1 (CFRT1,7 fractions, 14 Gy, Jul-Aug 2025) was ineffective (PD), while Radiotherapy Phase 2 (HFRT + Anlotinib, Aug-Sep 2025) achieved a PR. Radiotherapy Phase 3 (CFRT2, 12 fractions, 24 Gy) maintained remission. *Anlotinib was discontinued due to grade 3 cutaneous adverse effects.

A 59-year-old female professor specializing in basic medicine serves as the patient, with no reported family background of BC, smoking habits, or alcohol use. She indicated the absence of other significant chronic conditions, including hypertension, diabetes, or heart disease. At the time of treatment, her performance status was classified as ECOG 1, signifying her ability to manage self-care and engage in light physical activities, which is consistent with her capacity to endure intensive multiline therapies. Her educational and professional experience likely enhances her understanding, involvement, and adherence to the treatment plan, and her overall psychosocial condition was evaluated as positive. In July 2023, she discovered a mass in the upper outer quadrant of her left breast, roughly the size of a table tennis ball. The mass felt firm and was moderately mobile, with no associated breast pain, skin alterations, nipple retraction, nipple discharge, or systemic symptoms present. Following this, she had a mammogram conducted at Mount Sinai Hospital in the United States, which verified the existence of a breast mass. In April 2024, she was referred to Harbin Cancer Hospital, where an ultrasound-guided fine-needle aspiration biopsy was conducted. The biopsy findings revealed grade III invasive ductal carcinoma in the left breast, with involvement of the epidermis and the presence of vascular tumor emboli. Prior to surgery, the patient received a four-cycle systemic therapy regimen known as the CAB regimen. On day 1, she received an intravenous dose of Toripalimab (200 mg); on day 2, an intravenous dose of Abraxane (300 mg); and on day 3, an intravenous dose of Carboplatin (500 mg), with treatments taking place every three weeks (Q3W) (refer to **Figure 1**).The treatment plan was then modified to the AC regimen, which included an intravenous administration of 20 mg Liposomal Doxorubicin on the first day, followed by 40 mg on the second day, along with an intravenous dose of 1000 mg Cyclophosphamide on the first day, repeated Q3W for a total of three cycles. After the final administration of the AC regimen, the patient underwent a modified radical mastectomy in mid-October 2024. Pathological examination post-surgery confirmed invasive ductal carcinoma grade III in the left breast, measuring 4.0 × 4.0 × 3.8 cm, with negative margins, positive findings in 3 out of 18 axillary lymph nodes, and evidence of lymphovascular as well as perineural invasion. The immunohistochemical analysis revealed: ER -, PR -, HER2 positive at 2+, Ki-67 at 50%, and P53 negative. Following this, the fluorescence *in situ* hybridization (FISH) assay was optimized and conducted per the manufacturer’s specifications using the HER2 Spectrum Orange/CEP17 Spectrum Green probe (PathVysion HER-2 DNA Probe Kit; catalog number 02J01-030; Abbott Molecular Diagnostics), demonstrating HER2 amplification consistent with the standards set by the American Society of Clinical Oncology.

**Figure 2:**
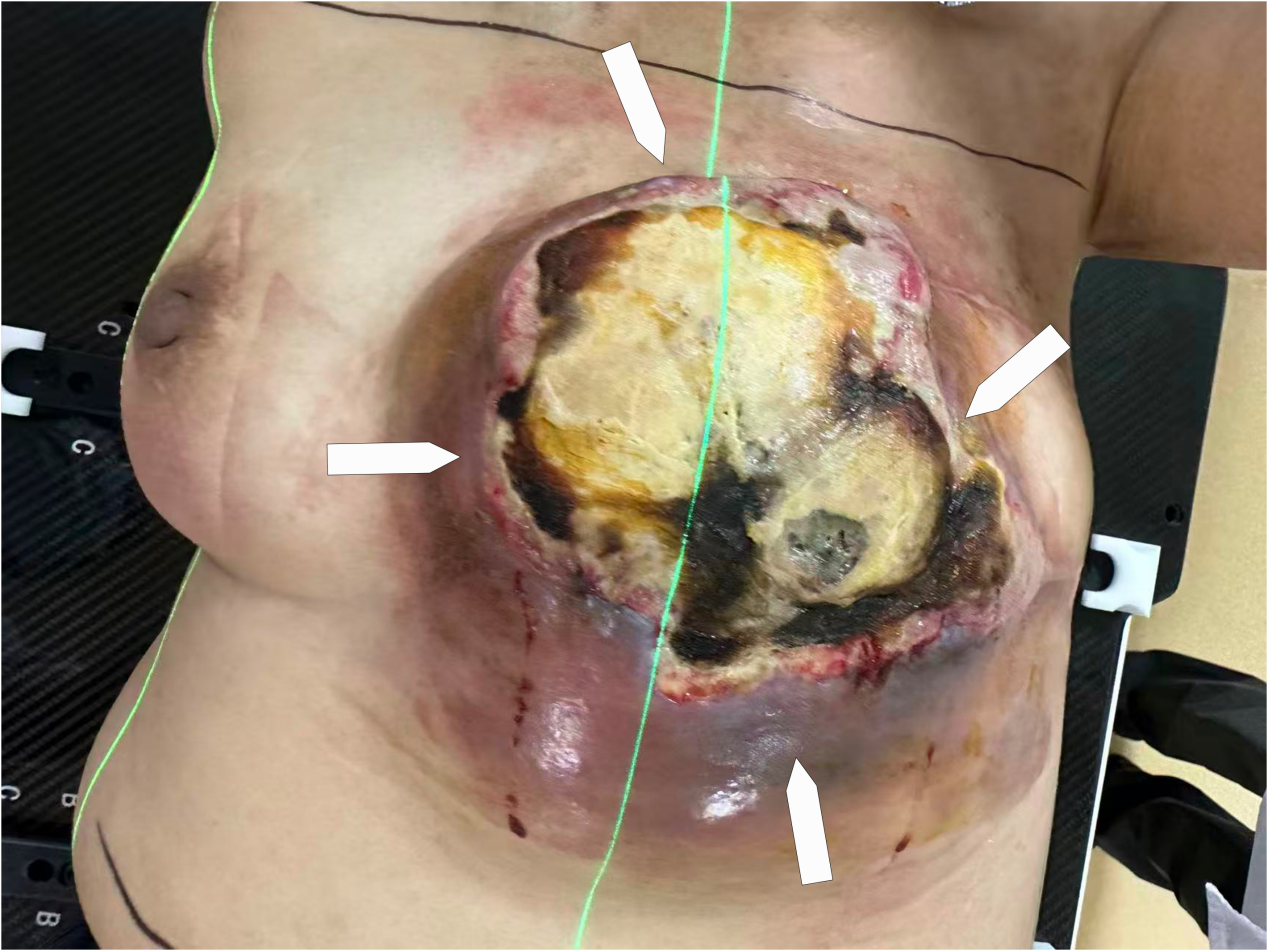
Clinical appearance of the left chest wall lesion. A large, protruding mass is observed in the presternal area, measuring approximately 16.5 × 14.3 × 6.8 cm (demarcated by four white arrows). The lesion exhibits significant ulceration with necrotic tissue and a pale yellow serous discharge. The surface shows an irregular, infiltrative border with a purple hue and prominent telangiectasia. On palpation, the mass is solid, fixed to underlying tissues with poorly defined margins.

In January 2025, the patient noted the development of a mass, roughly the size of a thumb, located in the presternal area, which was hard and exhibited restricted mobility. By February, this mass had progressively grown to about the dimensions of a ping-pong ball. Considering the patient’s prior history of a modified radical mastectomy due to left BC, the foremost diagnostic concern is CWR, which stands as the most prevalent type of local recurrence after BC surgery. Nevertheless, other potential diagnoses must be taken into account, such as benign soft tissue tumors post-surgery (like keloids or lipomas) or lesions from skin appendages. The mass’s clinical features—specifically its firm consistency and restricted movement—strongly indicate malignancy. Although imaging tests can offer important insights, they do not conclusively determine the lesion’s nature; hence, acquiring pathological tissue is essential for developing accurate future treatment strategies, especially regarding targeted therapies. As a result, a needle biopsy was conducted at Mount Sinai Hospital in the United States, and the pathology findings ultimately verified the diagnosis of recurrent BC in the chest wall. The immunohistochemistry findings were as follows: ER -, PR -, HER2 3+, Ki-67 index at 60%, and PD-L1 negative (CPS = 0) as determined by the anti-PD-L1 clone 22C3. Following this, the patient commenced targeted therapy using Trastuzumab (296 mg, intravenously, every three weeks) for a maintenance regimen lasting eight cycles. However, due to poor tumor response, a CT scan conducted in late July 2025 confirmed an increase in the tumor size to 16.5 × 14.3 × 6.8 cm, accompanied by skin ulceration and exudation. Additionally, a right axillary lymph node measuring 6.7 × 6.4 cm was observed(refer to **Figure 2**). It is worth noting that although the patient’s HER2 status was assessed, comprehensive next-generation sequencing (NGS) was not performed after disease progression to explore possible resistance mechanisms related to Trastuzumab resistance.

Subsequently, the patient was referred to our hospital (The Second Affiliated Hospital of Harbin Medical University) for PET-CT imaging. The scan revealed a large soft tissue mass in the pre-sternal region and multiple subcutaneous nodules on the anterior and left chest walls, all with increased FDG uptake. Additional nodules were observed in the anterior mediastinum, pericardium, and above the diaphragm, as well as in both axillae. Multiple nodules on the left pleura were also noted, along with left pleural effusion and incomplete expansion of the lower lobe of the left lung. These findings were consistent with metastasis.

**Figure 3:**
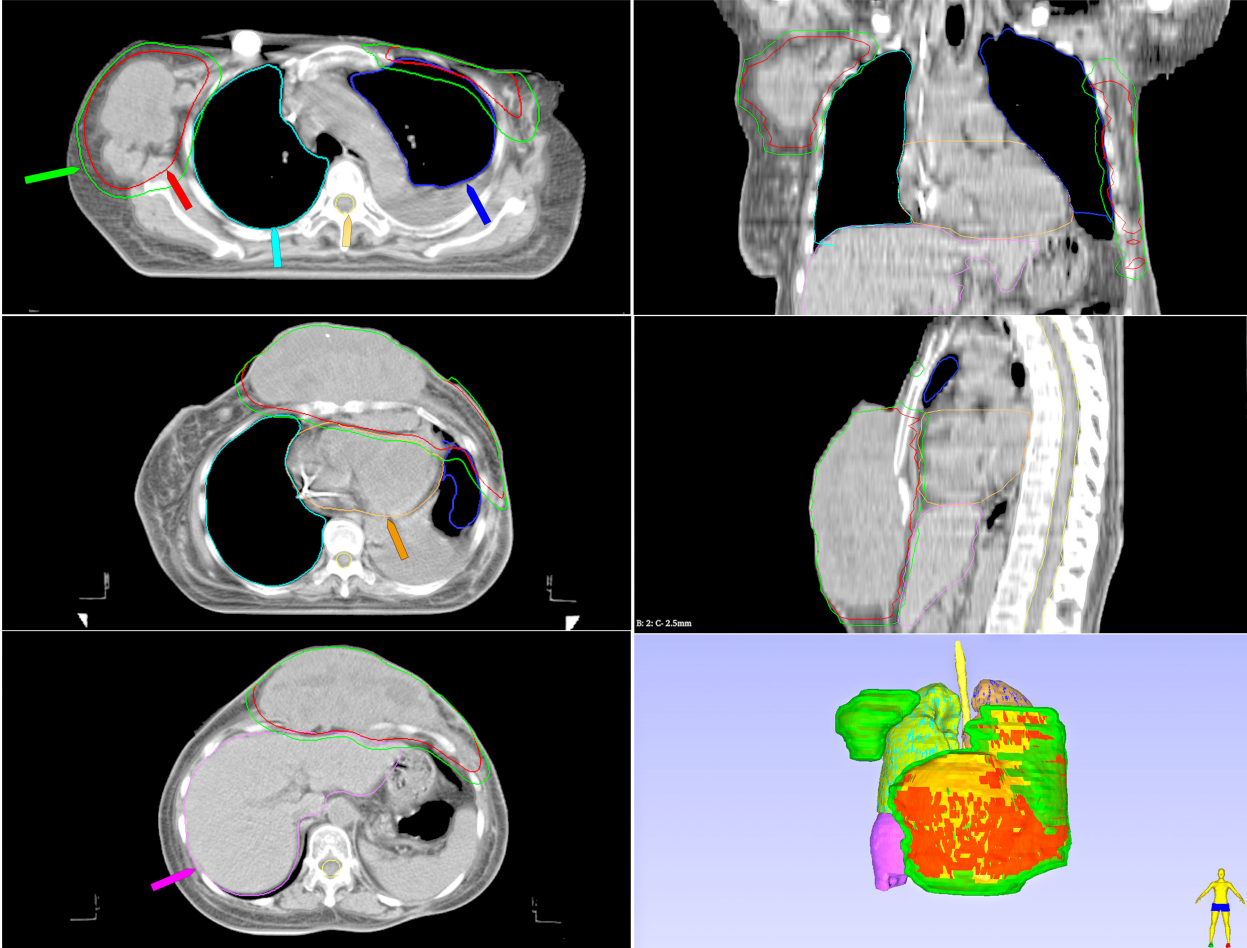
Radiotherapy target areas of CFRT1. The clinical target volume (CTV), encompassing the chest wall mass and axillary lymph nodes, is delineated in red. The planning clinical target volume (PCTV), generated by a 5 mm isotropic expansion of the CTV to account for setup and organ motion uncertainties, is shown in green. The OARs are color-coded as follows: right lung (light blue), left lung (dark blue), liver (purple), spinal cord (yellow), and heart (orange). The 3D view (bottom right) provides a comprehensive overview of the spatial relationship between the PCTV (green) and the surrounding anatomy.

Surgical intervention is not advised due to the engagement of the pleura and the presence of several metastases. For the unresectable recurrent mass situated in the chest wall, including about 1 cm of chest wall skin, radiotherapy is recommended. After undergoing modified radical mastectomy for breast cancer, the patient developed CWR, accompanied by skin ulceration and exudation. Given these factors, CFRT, as recommended by the National Comprehensive Cancer Network(NCCN)guidelines, was selected as the standard treatment. This approach is effective in eliminating potential residual microscopic cancer cells post-surgery, reducing the risk of recurrence, and is associated with relatively manageable side effects. Therefore, she was scheduled to receive CFRT with a dose of 2 Gy per fraction. To begin, a non-contrast free-breathing CT scan was conducted with a slice thickness of 2.5 mm. During the procedure, the patient was placed in a supine position with both arms lifted above the head, using a “breast board” as part of the immobilization setup. Chest computed tomography (CT) planning was done on a Somatom Emotion 16 scanner (Siemens Healthineers, Erlangen, Germany). The treatment plan was formulated utilizing Eclipse 15.6 (Varian Medical Systems, Palo Alto, California, USA), with the clinical target volume (CTV) and organs at risk (OAR) outlined on each CT scan(refer to **Figure 3**). The CTV included the full breast, chest wall, and right axillary lymph nodes. Contouring was carried out following the guidelines set by the NCCN. The clinically significant organs at risk identified comprised the lungs, heart, contralateral breast, spinal cord, esophagus, larynx, thyroid, and humeral head. Dose constraints for all OARs adhered to QUANTEC guidelines, with detailed dosimetric parameters presented in **Table 1**. The CTV was expanded by 5 mm to form the Planning Target Volume (PTV), which was treated with 6 MV photon intensity-modulated radiation therapy (IMRT). The radiotherapy prescription aimed to ensure that at least 95% of the PTV received the prescribed dose while strictly adhering to all OAR dose constraints. This approach utilized five evenly spaced coplanar beams delivered by a linear accelerator (linac; Varian DHX MLC, Varian Medical Systems), along with a 0.5 cm tissue compensation bolus to enhance the dose to the skin. All subsequent radiotherapy fractions were delivered under free breathing. This approach was chosen to ensure patient comfort (ECOG 1), and its safety was confirmed by dose-volume analysis of OARs (**Table 1**).

**Table 1:** Comprehensive dose analysis for target volumes and organs at risk.

Type	Organ/Target volume	CFRT1 (cGy)*	HFRT (cGy)*	CFRT2 (cGy)*	Cumulative Dose (cGy)	Cumulative Dose (Gy)	BED (Gy)
Target volume							
	CTV	4236.2	1550.6	3123.3	8910.1	89.1	83.25
	PCTV	4193.4	1535.5	3093	8821.9	88.2	82.4
Organ at risk							
	Heart	1497.5	566.9	1141.9	3206.3	32.1	55.8
	Lungs (Both)	1079.4	380.7	766.7	2226.8	22.3	38.8
	Left Lung	1685.7	660.3	1329.9	3675.9	36.8	64
	Right Lung	749.7	297.8	599.9	1647.4	16.5	28.7
	Liver	713.7	233.3	470	1417	14.2	24.7
	Kidney	141.7	54.1	108.9	304.7	3	5.2
	Spinal Cord	303.8	81.4	164	549.2	5.5	9.6
	Larynx	97.4	48.5	97.6	243.5	2.4	4.2

*Dose values are presented as mean.

The CTV received a high cumulative dose (89.1 Gy; BED = 83.25 Gy), sufficient for excellent local control. All OARs were spared, with doses well below established clinical tolerance thresholds. For instance, the doses to the heart (32.1 Gy; BED = 55.8 Gy) and spinal cord (BED = 9.6 Gy) indicate a very low risk of long-term toxicity. BED was calculated using an α/β ratio of 4 for target volumes and 3 for OARs.

Following a series of seven treatments amounting to 14 Gy, CT scans indicated a noticeable increase in the size of the patient’s chest wall mass and the right axillary lymph nodes, which measured roughly 18.2 x 17.4 x 8.2 cm and 9.1 x 8.3 x 8.5 cm, respectively, with no observed reduction. Unfortunately, new suspicious nodules were identified in the right chest wall, indicating disease progression according to RECIST v1.1 criteria. Given that CFRT was ineffective in controlling tumor growth and continuing with the same regimen could lead to increased toxicity, a switch to HFRT was considered. HFRT, with its higher per-fraction dose, offers more efficient tumor control in a shorter duration, minimizing prolonged radiation exposure and associated side effects, making it especially suitable for rapidly progressing tumors requiring urgent intervention. Furthermore, Anlotinib was introduced to improve tumor vasculature and reduce hypoxia, thus enhancing radiosensitivity. Considering the tumor’s progression, the risk of toxicity, the expected radiosensitizing effect of Anlotinib, and treatment timelines, a combination of HFRT and Anlotinib was selected.

**Figure 4:**
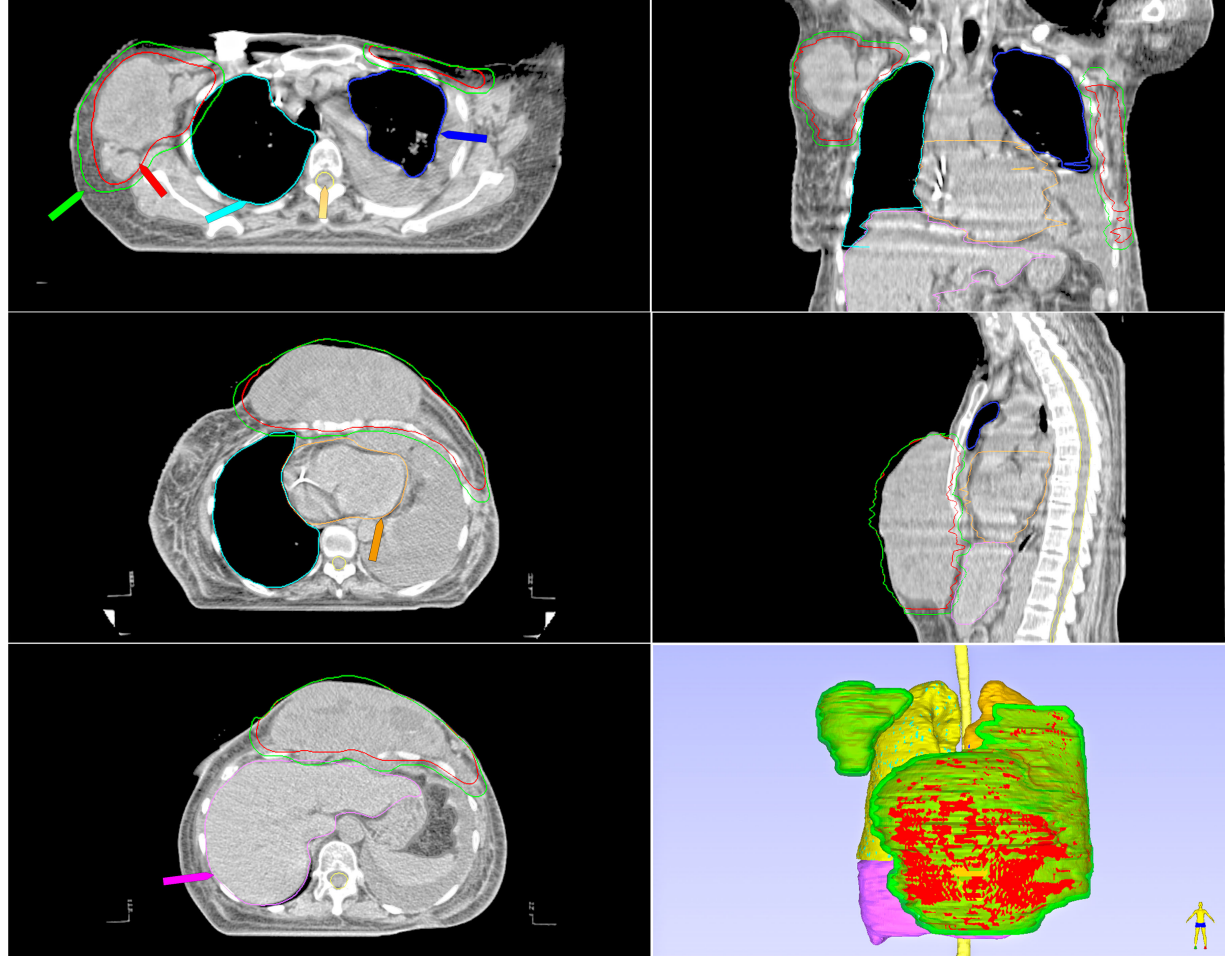
Radiotherapy target areas of HFRT and CFRT2. Multiplanar and 3D views display the CTV( red) and PCTV( green), along with color-coded OARs. The CTV, which includes the chest wall mass and axillary nodes, is significantly larger than in **Figure 3**, confirming PD.

As a result, the treatment area was redefined(refer to **Figure 4**), leading to the creation of a radiotherapy plan that involved HFRT delivering 15 Gy across five fractions, alongside Anlotinib treatment (a tyrosine kinase inhibitor given at 12 mg per dose for two consecutive weeks, followed by a one-week break). The radiotherapy led to local grade 3 moist desquamation as a complication. Importantly, following five HFRT sessions, there was a notable decrease in the size of the tumor lesions. Written informed consent was secured for the combination approach involving anti-angiogenic drugs together with HFRT and CFRT. HFRT promotes tumor cell death through high-dose single-fraction treatment, while CFRT enhances this effect over a prolonged period with lower doses, gradually eliminating residual tumor cells. Additionally, certain tumors may become more responsive to radiotherapy due to changes in the tumor microenvironment, such as vascular remodeling or reduced hypoxia. Therefore, CFRT and Anlotinib was selected for subsequent treatment, as it minimizes toxicity to normal tissues while continuing to effectively control the tumor. The final session of radiotherapy was completed on August 29, with a total of 12 CFRT sessions. These 12 CFRT sessions represent the third phase of treatment, following the completion of the initial two phases (seven CFRT sessions and five HFRT sessions). This sequential radiotherapy plan, carried out in three phases, has a total cumulative dose and biologically effective dose (BED) outlined in **Table 1**, with the detailed treatment schedule provided in **Table 2**.

**Table 2:** Sequential treatment timeline and clinical rationale.

Phase	Timeline	Treatment regimen	Clinical trigger / rationale for transition	Tumor response
Phase 1	July 25–August 4, 2025	CFRT1	NCCN guidelines	Progressive Disease (PD)
Phase 2	August 7–13, 2025	HFRT + Anlotinib	Tumor progression Radiosensitizing effect of Anlotinib	Partial Response (PR)
Phase 3	August 14–29, 2025	CFRT2	Tumor response Toxicity	Partial Response (PR)

Phase 1 (CFRT1): Initiated per NCCN guidelines for bulky recurrence. The transition to Phase 2 was prompted by PD, with the rationale being the need for urgent intervention using HFRT for rapid tumor control and Anlotinib for expected radiosensitization to overcome radioresistance. Phase 2 (HFRT + Anlotinib): Designed for rapid tumor control via radiosensitization. The transition to Phase 3 was driven by a achieved PR and the need to consolidate this gain while minimizing long-term toxicity. Phase 3 (CFRT2): Maintenance therapy to consolidate response, further reducing tumor size to 15.2×13.4×5.5 cm.

At the conclusion of the entire radiotherapy course, a reduction in the size of the chest wall tumor was observed, with the lesion turning dark in color, accompanied by minor ulceration and exudation. The axillary lymph nodes were palpable, showing a decrease in size, with no new lymph nodes detected. CT imaging confirmed that the chest wall tumor had reduced to approximately 15.2 × 13.4 × 5.5 cm, and the axillary lymph node measured 7.1 × 6.3 × 6.8 cm, both showing significant shrinkage, indicating partial remission. There have been no reported adverse effects such as hypertension, hand-foot syndrome, hypertriglyceridemia, hypercholesterolemia, or hypothyroidism, and the treatment has been tolerated well. An imaging evaluation is scheduled for December 2025 to evaluate the tumor’s response. The patient developed a grade 3 cutaneous adverse reaction following one cycle of Anlotinib treatment, which necessitated the discontinuation of the drug. She described the reaction as ‘an extensive rash covering over 30% of the body surface area,’ which not only caused physical discomfort but also significantly affected her quality of life. After active symptomatic and supportive treatment, the rash improved to grade 2 severity. At this juncture, we engaged in comprehensive communication and risk assessment with the patient. As a professor of basic medical sciences, she exhibited a profound understanding of the treatment regimen. After carefully weighing the potential risks of severe adverse reactions from continued medication against the uncertain systemic benefits, she autonomously decided to discontinue Anlotinib, based on her personal considerations regarding quality of life. We fully respect and support this decision, which aligns with her personal values and right to self-determination.

## Discussion

Trastuzumab has shown considerable effectiveness in the treatment of HER2-positive BC. Nonetheless, research reveals that around 20-30% of patients with HER2-positive tumors develop resistance to Trastuzumab (23). For individuals with recurrent metastatic HER2-positive BC, especially those who have had inadequate responses to various systemic therapies—including those targeting HER2—establishing a suitable treatment plan poses a considerable dilemma. Moreover, fluctuations in HER2 expression levels often correlate with worse survival rates, making it harder to pinpoint the best systemic treatment approach, whether in adjuvant settings or in the care of recurrent and metastatic conditions (24, 25). Patients identified as high risk for recurrence should follow standard protocols involving radiotherapy to the chest wall or breast and regional lymph nodes post-mastectomy or after breast-conserving surgery. Although the effectiveness of radiotherapy is well-established, the ideal regimen remains debated. While CFRT continues to be a prevalent choice for targeting the chest wall and regional nodes. Current clinical and radiobiological evidence suggests that HFRT may be more beneficial, as BC cells could be more responsive to intensified doses per fraction (26). In trials evaluating HFRT, the majority of participants underwent whole breast irradiation (WBI), resulting in limited data concerning chest wall and regional nodal irradiation (RNI). Additionally, when breast reconstruction occurs, HFRT could lead to significant treatment-related complications.

Anlotinib, an innovative oral tyrosine kinase inhibitor (TKI), successfully targets VEGFR, PDGFR, FGFR, and c-Kit, showcasing effectiveness in various solid tumors (27–29). While its main mechanism does not directly impact HER-2, its role in modifying the tumor microenvironment and its capacity to restrain cancer cell growth position it as a promising treatment alternative. Clinical investigations have suggested that Anlotinib might be effective in specific patients with HER-2-positive BC exhibiting well-characterized drug resistance. In recent years, the utilization of Anlotinib in metastatic breast cancer (MBC) has attracted considerable research interest. A key study revealed that Anlotinib inhibits the growth of MCF-7 BC cells and triggers apoptosis through the downregulation of TFAP2C (30). Additionally, various studies have shown that HER2 downstream signaling pathways, including PI3K/AKT and MAPK, may be activated during Trastuzumab therapy, contributing to drug resistance. This indicates that Anlotinib’s downregulation of TFAP2C might reduce the activity of HER2-mediated PI3K/AKT and MAPK pathways, consequently inhibiting the growth, migration, and invasion of cells resistant to Trastuzumab while inducing cell death *in vitro*. Nonetheless, this theory requires further validation through *in vivo* animal studies and confirmation in extensive clinical trials.

In this instance, the individual was found to have BC, exhibiting HER2 tumor expression levels of IHC (2+)/FISH positive at the time of initial diagnosis and IHC (3+) upon recurrence. BC treatment guidelines indicate that a Trastuzumab-based regimen is the recommended first-line approach for patients with HER2-positive recurrent metastatic BC who have not previously undergone adjuvant therapy with Trastuzumab (31, 32). However, despite undergoing eight cycles of Trastuzumab, the tumor markedly expanded in size, and there was no observable response to the initial systemic therapy for the recurrent condition. As a next step, the patient underwent CFRT targeting the chest wall; nevertheless, the tumor continued to be uncontrolled and showed signs of further growth. The tumor displayed resistance against several standard chemotherapeutic drugs, anti-HER2 antibodies, molecular therapies, and CFRT. Following administration of 15 Gy of HFRT, a significant decrease in tumor size was observed, suggesting an enhanced sensitivity to elevated radiation doses. The subsequent use of CFRT for maintenance therapy led to an additional reduction in tumor volume.

The research team suggested that the antitumor effectiveness of the combination therapy might originate from a dual action mechanism. On one side, it is thought that BC cells show increased sensitivity to higher doses of radiation per fraction in comparison to traditional dose fractionation methods. This claim is bolstered by radiobiological evidence; according to the linear-quadratic (LQ) model, tissues are divided into early- and late-responding categories. Tissues with a high α/β ratio (greater than 10 Gy) are identified as early-responding, characterized by rapid proliferation of cells, whereas those with a lower ratio are deemed late-responding (33). Withers noted that according to the LQ model in radiobiology, late-responding tissues display greater sensitivity to changes in fraction size, thereby laying the groundwork for the utilization of HFRT (34).According to the research carried out by Yarnold et al., the estimated α/β ratio for BC was determined to be roughly 4 Gy, which is within the range of 0.75 to 5.01 Gy. Conversely, the α/β ratio for healthy breast tissue is approximately 3 Gy, indicating that BC tissue shows a dose fractionation sensitivity that is comparable to that of normal tissues (35). This implies that HFRT potentially offers good tolerability and optimal control of the disease without significantly heightening adverse effects, thus providing enhanced benefits for patients with BC. Moreover, CFRT is based on the long-standing belief that BC cells are less responsive to fluctuations in dose per fraction compared to surrounding normal tissues. Nonetheless, there is evidence indicating that BC might be more responsive to alterations in dose per fraction relative to several other types of cancers. In addition, studies conducted in Thailand and India (36, 37) have supported this assertion.

On the other hand, Anlotinib has the potential to improve radiosensitivity via two distinct mechanisms: enhancing conditions in hypoxic environments and altering the levels of DNA repair proteins. In this situation, the patient showed resistance to various treatment options, which included chemotherapy, Trastuzumab, and standard fractionated radiotherapy. Following only one cycle of Anlotinib (12 mg for two consecutive weeks) in conjunction with five sessions of HFRT (totaling 15 Gy), notable shrinkage of the chest wall lesion was observed. This swift response pattern indicates that Anlotinib likely contributed significantly through a unique mechanism of radiosensitization, as opposed to merely depending on its systemic properties as an anti-angiogenic agent. It rapidly reorganizes the tumor’s microvascular architecture by inhibiting targets like VEGFR2 and PDGFRβ, thus mitigating tumor hypoxia and improving the effectiveness of radiotherapy in eliminating hypoxic cells (19). Additionally, prior studies have indicated that Anlotinib can decrease the levels of crucial DNA damage repair proteins (such as Rad51), extend the repair duration of radiation-induced DNA double-strand breaks, and thereby increase the cytotoxic effects of radiation (38). HFRT, by delivering a single higher dose (3 Gy per fraction), can more efficiently induce apoptosis in vascular endothelial cells and promote immunogenic cell death. When paired with Anlotinib’s influence on vascular normalization, this combination may create a beneficial feedback loop of “vascular targeting-radiosensitization”: Anlotinib lowers abnormal vascular density, while HFRT selectively targets and destroys remaining pathological blood vessels, concurrently releasing tumor antigens to stimulate local immune responses. This hypothesis aligns with the clinically noted phenomenon of rapid tumor regression but necessitates further investigation and confirmation through extensive clinical trials.

The significant therapeutic effectiveness noted in this instance, while promising, should be understood within the context of current literature. Existing randomized trial findings concerning HFRT for recurrent BC mainly emphasize WBI following breast-conserving surgery (10–13). There is comparatively limited data advocating for high-dose fractionation in chest wall and RNI (14). This case effectively utilized HFRT to treat unresectable CWR and showcased tumor responsiveness to high-dose fractions, which corresponds with established radiobiological principles (26). These observations offer important clinical evidence supporting the use of HFRT in this particular clinical situation. Furthermore, while evidence for Anlotinib’s efficacy in HER2-positive BC is still lacking, it has demonstrated significant antitumor activity and a favorable safety profile in various solid tumors, including non-small cell lung cancer (NSCLC) and small cell lung cancer (SCLC) (34, 35). These results underscore Anlotinib’s potential as a potent multi-targeted TKI. What distinguishes this case is our investigation into its innovative role in BC—as a radiosensitizing agent rather than just a systemic treatment. This approach has shown promise in studies combining other anti-angiogenic agents (like bevacizumab) with radiotherapy, and our findings provide initial clinical evidence for Anlotinib’s application in this domain.

Finally, we must emphasize four limitations of this study. Firstly, following eight cycles of Trastuzumab treatment, the patient experienced disease progression, possibly suggesting resistance to the drug. Nonetheless, there were no subsequent evaluations of the expression levels of genes tied to the HER2 signaling cascade, nor was there any genomic sequencing conducted on tumor tissue or circulating tumor DNA (ctDNA) to identify gene mutations or other genomic changes related to Trastuzumab resistance. Secondly, administering HFRT exclusively after CFRT may alter the biological characteristics of the tumor, potentially impacting the effectiveness of HFRT. This approach could also result in an increased radiation dose in normal tissues, thereby elevating the risk of injury to adjacent healthy tissues. Thirdly, the effectiveness of Anlotinib for HER2-positive BC is yet to be thoroughly investigated. And the patient’s choice to stop Anlotinib after just one cycle of treatment, without any follow-up maintenance therapy, might influence future treatment options. Lastly, as a single-case report, the findings may be influenced by patient-specific factors, such as unique tumor biology, which could introduce potential bias. Therefore, despite the promising nature of these results, there is an urgent need for rigorously designed prospective clinical trials to validate these findings further. Furthermore, these findings require rigorous long-term follow-up to confirm survival benefits and monitor for late toxicities.

In conclusion, this report presents a case involving recurrent HER2-positive BC where the disease progressed after treatment with Trastuzumab. The condition was later effectively managed through a combined treatment approach using Anlotinib and HFRT. The results from this case study indicate that administering Anlotinib alongside HFRT could offer clinical advantages for patients suffering from recurrent HER2-upregulated BC. This is particularly true for those with large, unresectable chest wall lesions that have not responded to previous treatments, including Trastuzumab. Nonetheless, further validation via prospective clinical trials is necessary to support this conclusion.

## Data Availability

The original contributions presented in the study are included in the article/supplementary material. Further inquiries can be directed to the corresponding author.
